# Altered Actin Centripetal Retrograde Flow in Physically Restricted Immunological Synapses

**DOI:** 10.1371/journal.pone.0011878

**Published:** 2010-07-29

**Authors:** Cheng-han Yu, Hung-Jen Wu, Yoshihisa Kaizuka, Ronald D. Vale, Jay T. Groves

**Affiliations:** 1 Research Centre of Excellence in Mechanobiology, National University of Singapore, Singapore, Singapore; 2 Department of Chemistry, University of California, Berkeley, California, United States of America; 3 Physical Biosciences and Materials Sciences Divisions, Lawrence Berkeley National Laboratory, Berkeley, California, United States of America; 4 Department of Cellular and Molecular Pharmacology, University of California San Francisco, San Francisco, California, United States of America; 5 Biomaterials Center, National Institute for Materials Science, Tsukuba, Ibaraki, Japan; 6 Howard Hughes Medical Institute, Chevy Chase, Maryland, United States of America; New York University, United States of America

## Abstract

Antigen recognition by T cells involves large scale spatial reorganization of numerous receptor, adhesion, and costimulatory proteins within the T cell-antigen presenting cell (APC) junction. The resulting patterns can be distinctive, and are collectively known as the immunological synapse. Dynamical assembly of cytoskeletal network is believed to play an important role in driving these assembly processes. In one experimental strategy, the APC is replaced with a synthetic supported membrane. An advantage of this configuration is that solid structures patterned onto the underlying substrate can guide immunological synapse assembly into altered patterns. Here, we use mobile anti-CD3ε on the spatial-partitioned supported bilayer to ligate and trigger T cell receptor (TCR) in live Jurkat T cells. Simultaneous tracking of both TCR clusters and GFP-actin speckles reveals their dynamic association and individual flow patterns. Actin retrograde flow directs the inward transport of TCR clusters. Flow-based particle tracking algorithms allow us to investigate the velocity distribution of actin flow field across the whole synapse, and centripetal velocity of actin flow decreases as it moves toward the center of synapse. Localized actin flow analysis reveals that, while there is no influence on actin motion from substrate patterns directly, velocity differences of actin are observed over physically trapped TCR clusters. Actin flow regains its velocity immediately after passing through confined TCR clusters. These observations are consistent with a dynamic and dissipative coupling between TCR clusters and viscoelastic actin network.

## Introduction

The formation of an immunological synapse, the intercellular junction between T cells and antigen presenting cells (APCs), involves formation of intermembrane protein complexes and micrometer length scale lateral reorganization within the interface [1], [2], [3]. For example, T cell receptor (TCR) engages the antigenic peptide loaded major histocompatibility complex II (pMHC) on the APC and forms sub-micrometer clusters during synapse formation. TCR clusters recruit various cytoplasmic molecules, such as LAT, Zap70, and SLP76, which carry out downstream T cell signals [4], [5], [6], [7]. Under sufficiently high antigen stimulation, TCR clusters subsequently move inward to the center of the junction, forming the central supramolecular adhesion complex (c-SMAC). Physically interfering with their transport process has been shown to affect both TCR-specific tyrosine phosphorylation and intracellular calcium flux [8]. Thus forced movement of TCR clusters, driven from within the cell or externally, can alter TCR signaling behavior.

For decades, the dynamic actin network and related proteins have been known to regulate various cellular processes, including cell migration, membrane protrusion, focal adhesion [9], [10], and aspects of T cell immunological synapse formation. Previous live T cell studies indicate that radial-symmetric actin filaments polymerize at the lamellipodia, subsequently flow back through the lamella, and depolymerize further towards the center of synapses [11], [12], [13]. Cortical actin centripetal retrograde flow provides an obvious candidate to drive radial protein sorting, and biochemical studies have shown that cytoskeletal drugs which disrupt actin polymerization can effectively inhibit immunological synapse formation [9], [13], [14]. Here, we utilize biophysical methods to directly investigate the associations of cortical actin retrograde flow and inward transport of TCR clusters.

Supported lipid bilayer membranes have been widely employed to manipulate membrane organization while preserving two-dimensional fluidity of the membrane [15], [16], [17]. Functional proteins can be stably incorporated into supported membranes, and these can form signaling interfaces with living cells. Micropatterned hybrid live cell - supported membrane junctions provide spatial control over the lateral transport of both intracellular and intercellular signaling molecules [8], [13], [18], [19], [20], [21]. Specifically, micro- or nanofabricated metal lines on glass substrates create barriers to lateral mobility in supported membranes and passively restrict molecular transports. Physically matching the metal line height to the supported membrane thickness and passivating by casein incubation minimizes non-specific interactions between the structured substrate and the cell.

Jurkat T cells transfected with EGFP-actin are triggered by mobile anti-CD3ε on supported membrane and utilized to visualize dynamical actin flow network in hybrid immunological synapses. Using this system, we are able to simultaneously visualize the dynamical reorganization of both TCR clusters and actin cytoskeleton in living Jurkat T cells. As ligated TCR clusters are spatially confined by barriers, it allows us to study localized biophysical interactions between actin centripetal retrograde flow and TCR clusters. In this work, we primarily focus on TCR coupling to actin network, and anti-CD3ε/TCR ligations are the only intermembrane linkages between T cell and supported membranes. By analyzing translocation trajectories of both TCR clusters and actin speckles, we demonstrate their non-static associations in triggered T cell synapses.

The centralized movements of TCR clusters are attributed to actin centripetal retrograde flows. Notably, modulations of actin flow velocity in repatterned immunological synapses are observed. We find that actin flow velocity distribution is significantly decreased in proximity of confined TCR clusters and then recovers to normal levels after traversing. The localized reduction of actin flow velocity by TCR clusters would be expected from a dissipative coupling [18] between TCR clusters and the viscoelastic actin network [22], [23], [24], [25], [26]. In contrast, a more static association between TCR and actin, as would be predicted if strong TCR-adapter-actin binding interactions existed, would be expected to result in trapped actin at fixed TCR clusters and flow patterns around the fixed obstacles. The ability to spatially restrict the movement of membrane-associated molecules creates more possibilities to biophysically examine dynamic associations of signaling molecules on the plasma membrane and cytoskeletal networks in various cellular responses.

## Materials and Methods

### Cell culture and reagents

Jurkat T cells are cultured in RPMI 1640 media and transfected with EGFP actin as described in [13]. 1,2-dioleoyl-sn-glycero-3-phosphocholine (DOPC) and biotinylated phosphatidylethanolamine with a caproyl spacer (biotin-CAP-PE) are purchased from Avanti (Alabaster, AL). Anti-CD3ε (Hit3a) monoclonal antibody is purchased from BD (San Diego, California) and then monobiotinylated following the previously described procedure [13]. Texas red-conjugated streptavidin is purchased from Invitrogen (Carlsbad, CA).

### Substrate fabrication

Glass substrates (#1 circular coverslip, Fisher Scientific, Pittsburgh, PA) are cleaned by sonication in isopropyl alcohol and water mixture (1∶1 volume), followed by piranha acid wash (3∶1 sulfuric acid and hydrogen peroxide) for 5 minutes. S1805 positive resist (Microchem, Newton, MA) is spun on substrates, exposed with UV light through designed photomasks, and developed. Next, 5 nm of chromium layer is deposited by an electron-beam thermal evaporator and patterned by a lift-off process in acetone in a sonication bath. Before usage, patterned substrates are treated with piranha to maintain hydrophilicity, washed thoroughly in deionized water, and dried under nitrogen gas.

### Supported membrane preparation

Small lipid vesicles (SUVs) with DOPC and 0.02 mole percent of biotin-CAP-PE are prepared as described [13]. SUVs are diluted into phosphate buffered saline solution and exposed to a hydrophilic patterned glass substrate to form a supported lipid bilayer membrane. Casein (Fisher Scientific, Pittsburgh, PA) is then used for blocking non-specific binding. Texas-Red streptavidin and monobiotinylated anti-CD3ε antibody are sequentially introduced and conjugated with biotinylated lipids in micropatterned supported membrane. Excess Anti-CD3ε is washed out before adding cells.

### Imaging

Two-color time-lapsed confocal microscopy is performed on a Nikon TE2000U inverted microscope with Yokogawa CSU22 spinning disk confocal scanner unit. Samples are excited by Ar (488 nm) and Ar/Kr (568 nm) lasers, and fluorescence images are captured by Photometrics Cascade II EMCCD camera via acquisition software μManager. Fluorescence images are taken within 10 minute after cell landing. All experiments are conducted in a controlled temperature (37°C) and humidity chamber equipped on the microscope.

### Tracking algorithms

Time-lapsed images of TCR cluster movement and actin centripetal retrograde flow are analyzed by Particle Tracker in ImageJ and custom code in Matlab. Actin speckles tracked in each frame are performed in two stages, (1) identifying the location of actin speckles; (2) creating the trajectories of actin speckles in the consecutive frames. Images are first convolved with a Gaussian filter to remove the signal noise. The possible locations of actin speckles are detected by searching local maxima of intensity across the cell. Multiple local maxima are usually observed in single actin speckle. To further select and refine the position of significant speckles, the vector fields of image intensity gradient are created. The location of significant speckle is identified at the position with low intensity gradient. We utilize gradient vector flow algorithm to obtain the smooth vector field of gray level images [18], [27], [28]. By detecting the gradient of intensity, the variation of background intensity across the cell would not affect the process of searching speckles. The insignificant objects are successfully removed by applying this routine.

After detection of speckle positions, the motions of speckles are tracked. The tracks are generated by linking nearest neighbor speckles between consecutive frames if the distance between two positions is less than the moving tolerance of speckle motions. The average size of speckles is used as the moving tolerance. The appearance, disappearance, merging, and splitting events of speckles are monitored. If the speckles at time t cannot be linked to any position at time t+1, the speckles are considered as disappearance at time t+1. On the other hand, new trajectories are classified if speckles at time t+1 are not linked to any position at time t. Merging and splitting events sometimes are observed in the area with dense actin meshwork. If multiple speckles at time t are linked to the same speckle at time t+1, the merging events are recorded. On the contrary, the splitting events are monitored if multiple speckles at time t+1 are linked to the same speckle at time t+1. All the trajectory information is stored in the array for velocity computation. More than 25000 track information could be analyzed in a single synapse. By selection of regions of interest, localized velocity and angular histograms of actin speckles are calculated from identified tracks. Averages of radial and angular velocity distribution are calculated accordingly with their standard error of mean.

## Results

### Spatial mutations

**Figure 1:**
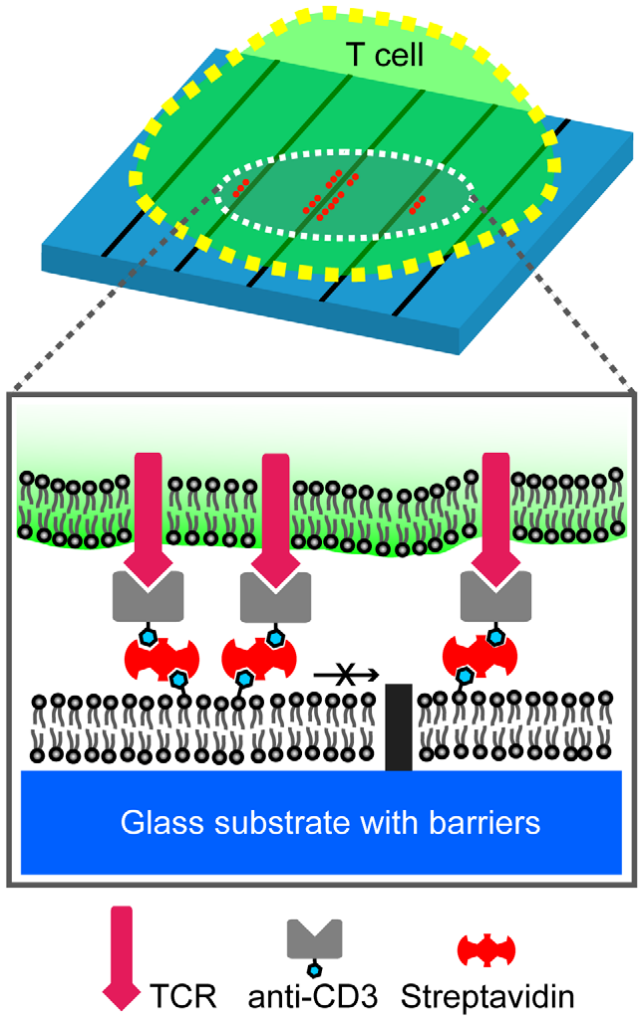
Experimental schematic. Monobiotinylated anti-CD3ε is attached to supported lipid membrane via biotin-streptavidin linkage. Jurkat T cell forms immunological synapses as TCR on T cell membrane engages with mobile anti-CD3ε on supported membrane. Microfabricated chromium stripes (0.5–1 µm in width, 5 nm in height, and 3–4 µm pitch) on glass substrates serve as diffusion barriers of TCR/anti-CD3ε clusters and limit the inward translocation of ligated TCR clusters. The confined TCR clusters along stripe patterns allow us to study their mechanical coupling with active transport mediated by cortical cytoskeleton.

**Figure 2:**
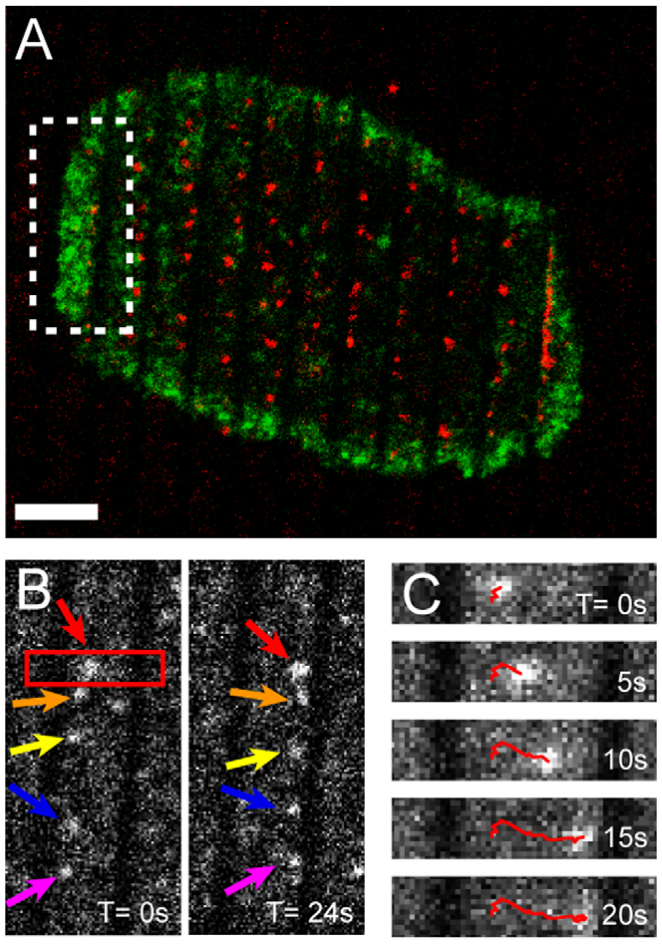
Spatially-mutated hybrid immunological synapse. (A) Fluorescence image shows that T cell with EGFP-actin (green) engages with micro-patterned substrates and triggers synapse formations. The spacing between each chromium stripe is 2 µm. Anti-CD3ε conjugated with Texas-Red streptavidin (red) is ligated with TCR on T cell membrane and forms submicrometer-sized clusters. (B) Inward movement of TCR clusters, a close-up view from the boxed region in (A). TCR clusters are nucleated and brought inwards from the periphery of the cell where the lamellipodium protrusions actively scan supported membrane surface. Diffusion barriers on supported membrane locally retain TCR clusters. See Movie S1 for detail demonstration. (C) Time-series images showing the single track from one group of TCR clusters, a close-up view from the boxed region in (B). Directional inward movement of TCR clusters suggests the underlying active transport process driven by actin centripetal retrograde networks. Scale bar 5 µm.

To generate spatial mutations, hybrid live T cell – supported membrane synapses are reconstructed over patterned glass substrates (Fig. 1). Parallel chromium lines of 0.5–1 µm in width, 5 nm in height, and 3–4 µm pitch are microfabricated on glass coverslips. Monobiotinylated anti-CD3ε is uniformly and stably linked to a supported lipid bilayer via biotin-streptavidin linkage. Live Jurkat T cells transfected with EGFP-actin are introduced after the supported membrane and anti-CD3ε are deposited. TCR on T cell membrane engages with mobile anti-CD3ε on supported membrane, form submicrometer-sized clusters with other signaling molecules, and then move towards the center of the synapse. Total 26 individual cells are examined. Microfabricated chromium stripe patterns partition the supported membrane and physically limit lateral translocation of ligated TCR clusters (Fig. 2 and Movie S1). Using spinning-disk fluorescence confocal microcopy, live actin centripetal retrograde flow is imaged (for the first time, to our knowledge) in micropatterned hybrid immunological synapses. The confined TCR clusters along stripe patterns allow us to study their mechanical coupling with actin cytoskeleton.

### Association of TCR clusters and actin centripetal flow

**Figure 3:**
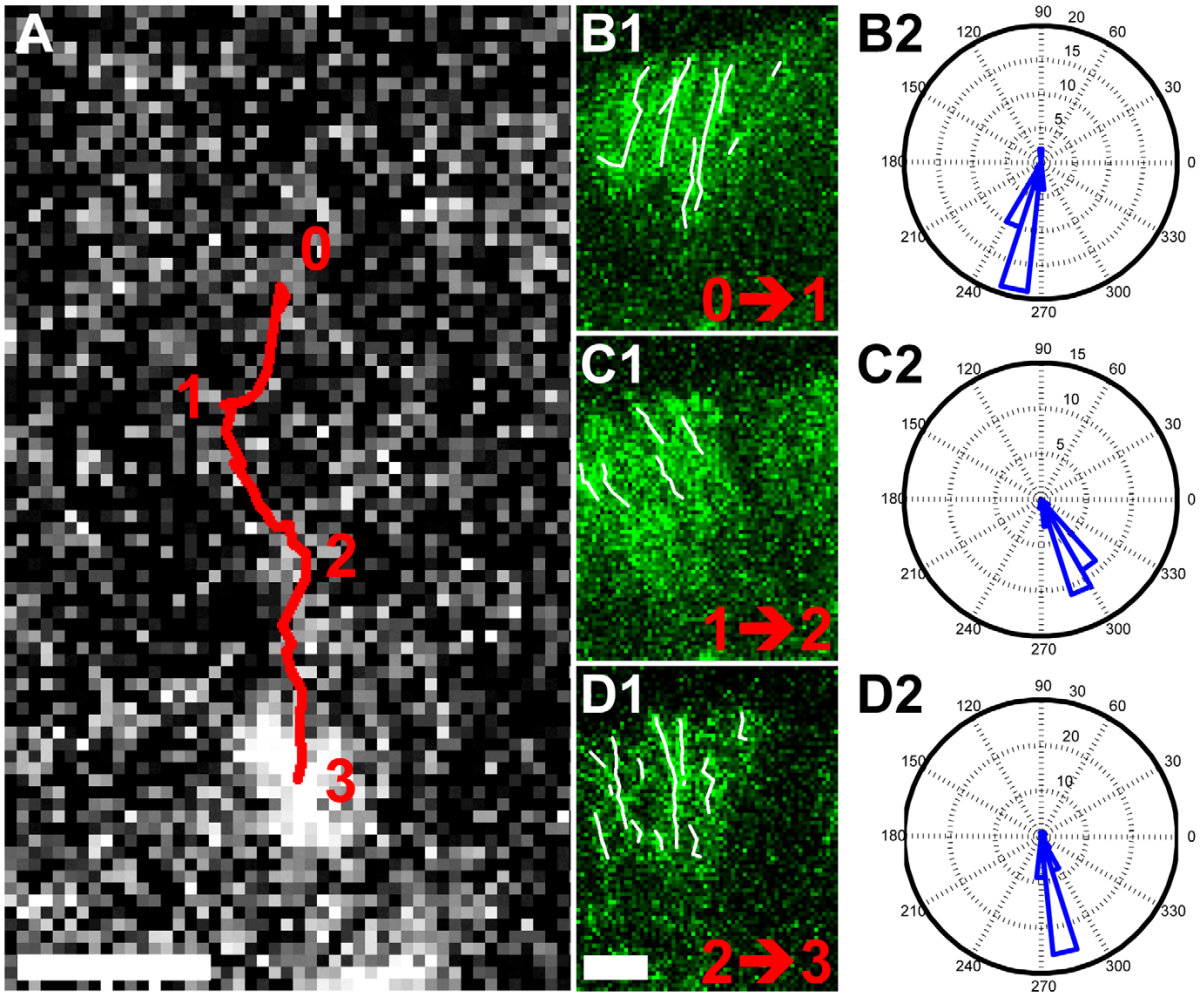
Dynamic association of TCR clusters and cortical actin flows. Confocal images of TCR clusters (A) and EGFP-actin speckles (B1, C1, D1) at the lamella in hybrid immunological synapse. Using particle tracking algorithms, the red track in (A) and white track in (B1, C1, D1) represents the translocation trajectories of TCR clusters and EGFP-actin speckles, respectively. TCR clusters behave non-diffusive motion and follow the directions of actin centripetal retrograde flow. From stage 0 to 1 (t = 0 to 9 sec), 1 to 2 (t = 10 to 24 sec), and 2 to 3 (t = 25 to 33 sec), cortical actin retrograde flow dynamically changes its direction and simultaneously regulates TCR cluster translocations with corresponding direction. (B2, C2, D2) Angle histograms of actin flow direction in different stages. Angle of movement is derived based on the starting point along each track. The histograms suggest that TCR and actin move in nearly identical directions within the same stage. See Movie S2 for detail demonstration. Scale bar 1 µm.

Previously, we have reported potential transient linkages between TCR clusters and cortical actin flow that collectively give rise to a frictional drag force to drive TCR cluster movement. Unlike distinct TCR tracks, actin networks are visualized in the present study as moving speckles. Low concentrations of EGFP-actin monomers generate heterogeneous labeling of actin filaments, which provides image constrast sufficient to track flows. With the ability to visualize actin dynamics using fluorescence confocal microscopy, we confirm that TCR clusters and actin centripetal retrograde flow concurrently move inward to the center of the synapse. As cortical actin constantly polymerizes at the lamellipodia, flows towards the center of synapses, and becomes depolymerized, TCR movement is directed by continuous actin flow in a non-static manner. Synchronized particle tracking of both TCR clusters and actin speckles shows that TCR clusters followed actin flow (Fig. 3 and Movie S2). From stage 0 to 1 (t = 0 to 9 sec), 1 to 2 (t = 10 to 24 sec), and 2 to 3 (t = 25 to 33 sec), cortical actin centripetal flow dynamically changes its direction and simultaneously regulates TCR cluster translocation with corresponding direction (Fig. 3B1, 3C1, 3D1). More specifically, we calculate the angle of movement based on the starting point along each track within the same stage. Angle histograms of each trajectory indicate that TCR and actin move simultaneously in nearly identical directions (Fig. 3B2, 3C2, 3D2). Kymographs of both TCR and actin tracks also reveal their spatial-temporal correlations. At each time point, intensity profiles (plotted in x-axis) along the track are assembled along y-axis in time-descending order (Fig. S1). This graphical representation also confirms the dynamic associations of TCR clusters and steady-streaming actin flow.

### Actin velocity analysis in spatially mutated synapses

**Figure 4:**
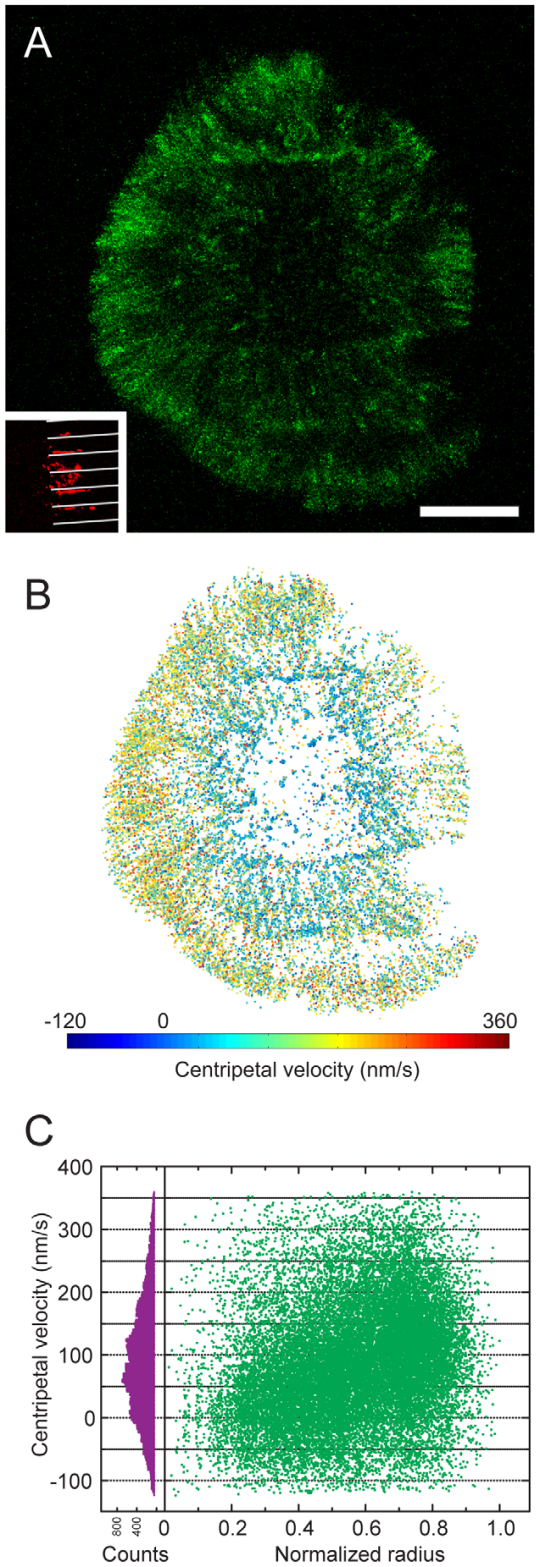
Actin velocity analysis in spatial mutated synapses. T cells with EGFP-actin are imaged by spinning disk confocal microscope while forming immunological synapses on micro-partitioned supported membranes. (A) Maximum intensity projection of EGFP-actin signals (green) over 100-second period. Cortical actin actively polymerizes at the lamellipodia and flows toward the center of synapse. See Movie S3 for detail demonstration. Inset: Fluorescence image of reorganized anti-CD3ε clusters (red). White stripes represent microfabricated diffusion barriers (3 µm line spacing) which laterally confine TCR clusters. (B) Actin velocity map in spatial-mutated synapse. Actin speckles are identified and linked using flow-based particle tracking algorithms. Actin flow velocity is calculated between adjacent points in each track. Color-coded velocity map shows that actin centripetal retrograde networks are locally modulated by retained TCR clusters. Negative radial velocity calculated from tracking algorithms generally contributes from intrinsic density fluctuation of actin speckles. (C) More than 25000 tracking information are analyzed, and mean velocity is 88.2±0.6 nm/s (SEM, *n* = 25083). Normalized histogram of entire measured actin centripetal velocity (left chart, purple) reveals wide spread of actin velocity. Spatial distributions of actin velocity are plotted with normalized radial positions (right chart, green). This also indicates general decay of flow velocity as actin moves further inwards. Scale bar 5 µm.

In spatially mutated synapses, we find both the cortical actin centripetal flow and TCR clusters can be reorganized (Fig. 4A and Movie S3). Microfabricated substrates locally restrict TCR clusters translocation (Fig. 4A inset) and show no direct perturbations to unligated molecules, as the height of chromium stripes on glass substrates is designed to match the thickness of the lipid bilayer (about 5 nm). Using flow-based tracking algorithms, actin speckles are effectively identified and connected as individual tracks. Centripetal velocity of actin flow is measured from adjacent points in each track. Over 25000 tracking data of EGFP-actin in time-lapsed confocal images are calculated. A color-coded velocity map demonstrates that cortical actin flow velocity is spatially modulated where confined TCR clusters are located (Fig. 4B). Centripetal actin flow velocity we measure is widely spread (Fig 4C, left chart) and ranges as fast as 360 nm/s with mean velocity 88.2±0.6 nm/s (*n* = 25083, all errors are standard error of mean unless otherwise noted). Negative centripetal velocity calculated from tracking algorithms generally results from intrinsic density fluctuation of actin speckles. Centripetal velocity distribution of actin retrograde flow plotted with normalized radial positions also indicates general decay of flow velocity as actin moves further toward the center of synapse (Fig. 4C, right chart).

### Decrease of actin velocity when passing confined TCR clusters

**Figure 5:**
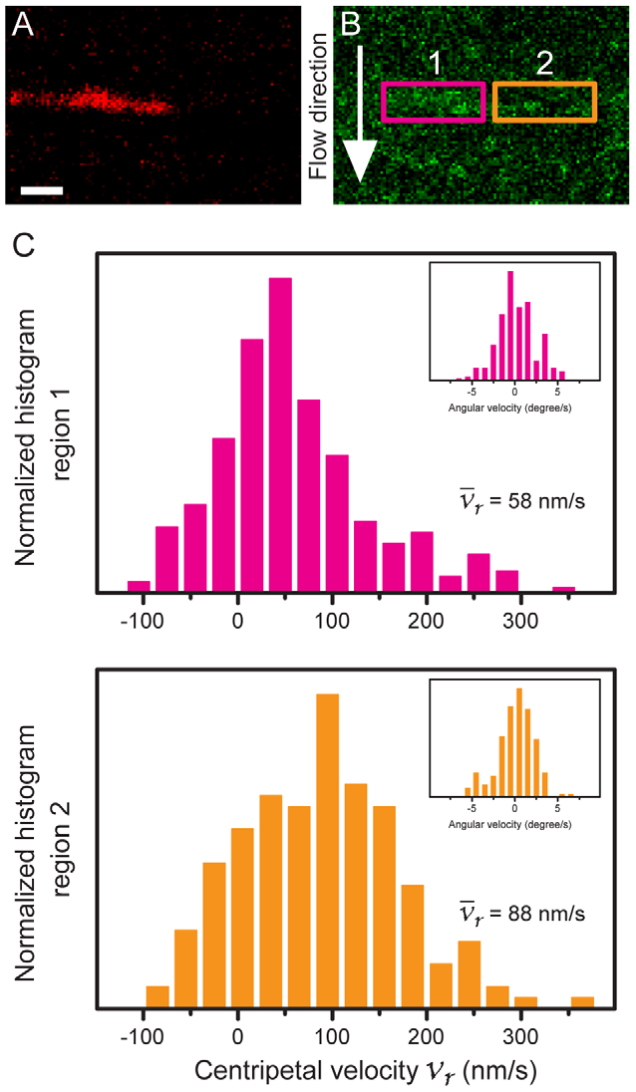
Decrease of actin velocity when passing confined TCR clusters. (A and B) Maximum intensity projections of confined TCR clustered (red) and EGFP-actin signals (green) over 45-second period. Region 1 and 2 are located in the lamella and have nearly identical radial distance to the center of synapse. TCR clusters in region 1 are spatially retained and accumulated against the chromium line perpendicular to actin flow. In region 2, no TCR buildup is observed along the same chromium line. (C) Actin velocity distributions inside region 1 and 2. More than 150 track information is analyzed in each region. Statistical distribution of radial actin velocity showed that actin flow in region 1 is slower, with the mean value of 58±4.9 nm/s (SEM, *n* = 166). In opposition, actin flow in region 2 is faster, with the average velocity of 88±6.5 nm/s (SEM, *n* = 269), and is closely comparable to the mean velocity value of bulk actin retrograde flow (Fig. 4D). This side-by-side comparison suggests that micropatterned substrates itself had no apparent influences on actin flow dynamics, but TCR clusters retained by microfabricated chromium lines does notably change local actin flow profiles. Scale bar 1 µm.

Detailed investigation of cortical actin flow dynamics reveals slower actin flow over confined TCR clusters whereas it stays the same level elsewhere. To perform localized velocity analysis, we examine actin velocity distributions in selected regions within the lamella in spatially-mutated immunological synapses (Fig. 5A and 5B). In particular, we choose two adjacent regions along a single chromium line, one with confined TCR clusters (region 1 in Fig. 5B) and one without (region 2 in Fig. 5B). Each selected region contributes more than 150 individual tracking information, and the statistical distribution of actin velocity shows that actin flow in region 1 of Fig. 5B (with confined TCR clusters) is slower, with the mean value of 58±4.9 nm/s (SEM, *n* = 166, Fig. 5C). Conversely, actin flow in region 2 of Fig. 5B (without TCR clusters accumulation) is faster, with the average velocity of 88±6.5 nm/s (SEM, *n* = 269, Fig. 5C). Angular velocity distribution (inset of Fig. 5C) suggests rather unidirectional actin flow in selection regions. The micropatterned substrate itself has no apparent influences on actin flow dynamics (Fig. S2), but TCR clusters retained by microfabricated chromium stripes notably changes local actin flow profiles. Hence, we directly observe the drag between the flowing actin network and the fixed TCR clusters.

### Actin flow regains velocity after passing confined TCR clusters

**Figure 6:**
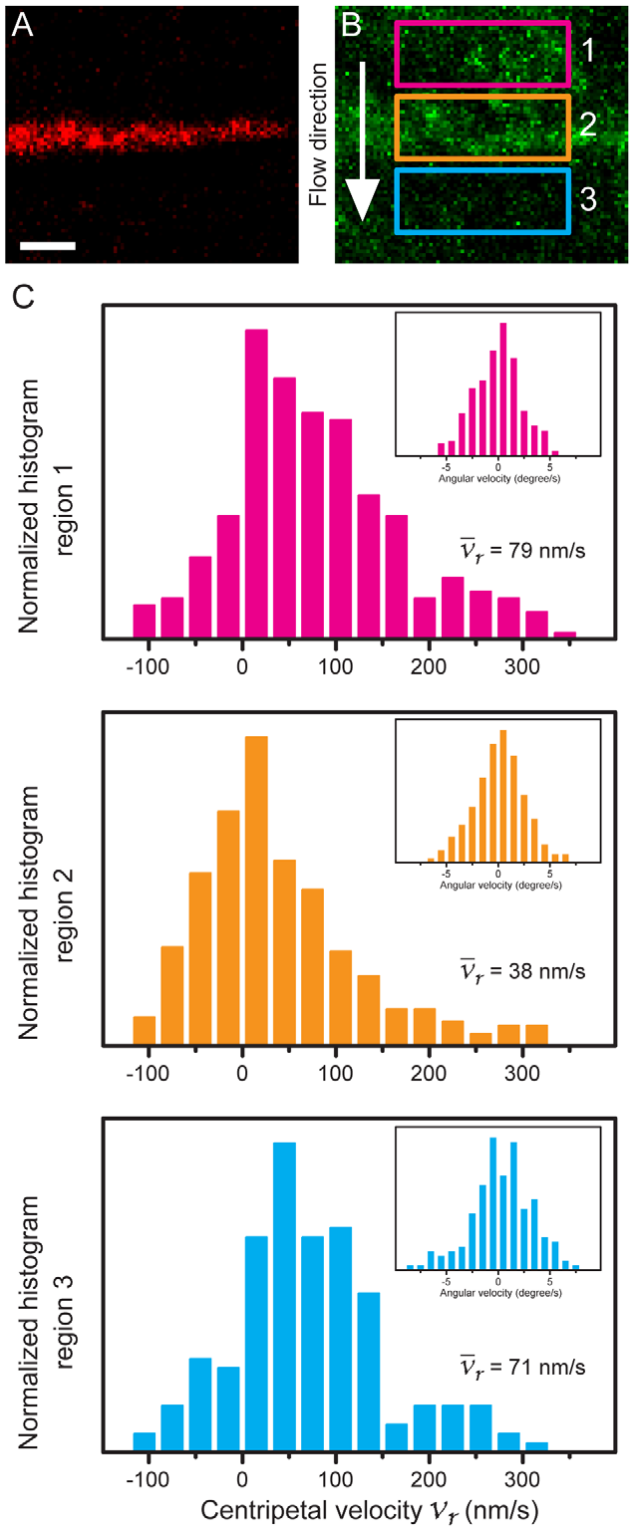
Actin flow regains velocity after passing confined TCR clusters. (A and B) Maximum intensity projections of confined TCR clustered (red) and EGFP-actin signals (green) over 45-second period. Actin speckles flow inwards and sequentially traveled through region 1, 2 and 3 consecutively within the lamella. TCR clusters in region 2 are spatially confined and accumulated against the chromium line perpendicular to actin flow while there are no TCR clusters accumulations in region 1 and 3. (C) Actin velocity distributions compared in those 3 regions. Each region includes more than 150 individual actin track information. Average radial velocity of actin flow in region 1, 2 and 3 are measured as 79±5.6, 38±4.4, and 71±6.3 nm/s, respectively (SEM, *n* = 261, 365, 167). The velocity decrease between region 1 and 2 confirms our previous result. Actin retains the velocity after passing region 2 where confined TCR clusters locate. The velocity recovery between region 2 and 3 suggests impermanent interactions between TCR clusters and cortical actin network. Scale bar 1 µm.

Next, we analyze three adjacent regions within the lamella along the direction of actin retrograde flow (Fig. 6A and 6B). Only one of the selected regions is in position with the confined TCR clusters (region 2 in Fig. 6B). Actin speckles flow inward and sequentially travel through region 1, 2 and 3 (Fig. 6B), and each region contains more than 150 individual tracking information. Localized actin velocity analysis in these three regions reveals differential velocity distributions along centripetal retrograde flow. Average velocities of actin flow in region 1 and 2 (Fig. 6B) are measured as 79±5.6 nm/s and 38±4.4 nm/s, respectively (SEM, *n* = 261 and 365, Fig. 6C). This velocity decline confirms our previous observation since region 2 (Fig. 6B) colocalized with confined TCR clusters. Notably, after passing confined TCR clusters in region 2, actin flow in region 3 regains the velocity, with the mean value of 71±6.3 nm/s (SEM, *n* = 167). The recovery of the actin velocity suggests impermanent and weak interactions between TCR clusters and the cortical actin network. The small velocity difference between region 1 and 3 results from the general actin velocity decay and depolymerization as it travels towards the center of synapses (Fig. 4D).

## Discussion

**Figure 7:**
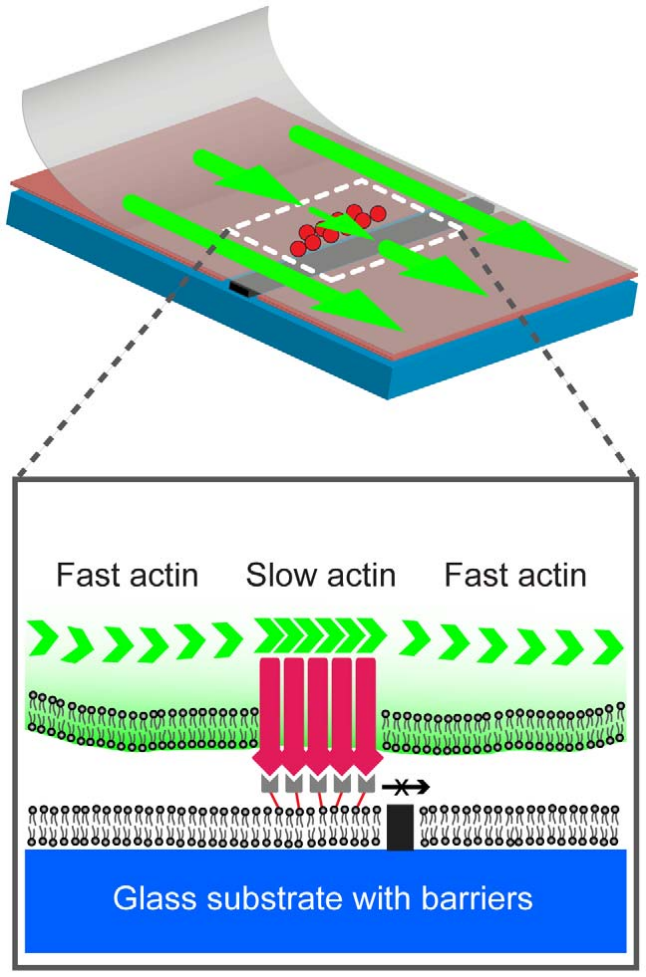
Frictional coupling model of TCR translocation and actin flow in immunological synapses. While the inward translocations of TCR clusters are dynamically directed by cortical actin centripetal retrograde networks, confined TCR clusters are also able to spatially modulate the velocity distributions of consequent cortical actin flow. Our results reveal the potential mechanism of force feedback interactions between signaling molecules on cellular membrane and cytoskeleton networks.

Based on our observations, a frictional coupling mechanism of TCR clusters and viscoelastic cortical actin network is proposed (Fig. 7). Previously, the dissipative coupling between TCR clusters and cytoskeletal network has been introduced [18]. Specifically, analysis of the deflections of TCR clusters motion by substrate-imposed barriers reveals a cosine scaling of the speed, with a deflection angle and no elastic memory of trajectory. Taken together these observations demonstrated that TCR clusters are not elastically coupled to the cytoskeleton, which itself does have an elastic component [22], [23], [24], [25], [26]. Here we directly demonstrate that actin centripetal retrograde flow serves as a mechanical driving force to transport TCR clusters. During the synapse formation, cortical actin continuously polymerizes in the lamellipodium at the periphery of the synapse and then actively moves inwards. TCR clusters that nucleate at the lamellipodium are found to be transiently associated with actin speckles and follow the direction of proximal actin centripetal flows.

Detailed analysis of actin speckles movement with micro-partitioned synapses enables us to investigate the spatial-temporal modulation of actin centripetal retrograde flow. Microfabricated chromium metal lines on the glass are around 5 nm in height (notionally lower than anti-CD3ε on lipid bilayer in height) and are passivated by casein incubation. These physical barriers of TCR cluster restrict translocations in immunological synapse and show no apparent effect on actin flow dynamics (region 2 in Fig. 5B and Fig. S2). Physically confined TCR clusters which consist of hundreds of TCR and associated adaptor proteins cooperatively contribute frictional drag onto adjacent actin retrograde flows [19], [29], [30]. This collective dissipative interaction effectively results in temporary decreases of actin flow velocity in the vicinity of confined TCR clusters.

The observation of actin flow velocity recovery after passing confined TCR clusters also implies viscoelastic properties of actin flow network. Theoretical optical slice thickness of spinning disk confocal microscopy is calculated from the equation [31],
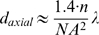
In our experiment setup, EGFP emission wavelength *λ_em_* = 509 nm, refractive index of aqueous media n = 1.33, and lens numerical aperture NA = 1.40, the axial resolution is approximately 484 nm. The thin confocal volume provided by spinning disk microscopy allows us to observe local remodeling of cortical actin networks. As negative velocities in the actin flow analysis are often contributed from density fluctuations of actin speckles within the confocal volume, the fast-moving actin speckles at spatially repatterned TCR clusters suggest structural mismatch of elastic and multilayered actin network (Fig. 5C and Fig. 6C). Proximal layers of actin flow network adjacent to confined TCR clusters are locally retained, and the bulk actin retrograde flows above continuously move inward. After traversing, slow-moving actin speckles rejoin overall actin retrograde network, possibly through nonmuscle myosin IIA linkage [32], [33] and then regain flow velocity.

While actin retrograde flow collectively directs the inward movement of TCR clusters, laterally-restricted TCR clusters (immobile) are also able to modulate the velocity distributions of consequent cortical actin flow via the frictional coupling model described above. We consider that these observations can be general biophysical phenomena in active molecular sorting on cellular membranes. Our results reveal the potential mechanism of force feedback interactions between signaling molecules on cellular membrane and viscoelastic cytoskeletal networks. Various biological events involve physical reorganization of molecules to amplify the signal and trigger the downstream pathways. Spatial partitioning of supported membrane provides a unique method to investigate intercellular molecule sorting and signaling by selectively introducing geometrical constraints to targeted intracellular membrane components. The ability to mechanically manipulate biophysical interactions between fluid plasma membranes (two-dimensional setting) and dynamical cytosolic protein assembly (three-dimensional environment) create more possibilities to study the underlying mechanism of mechano-transduction in living cells.

## Supporting Information

Figure S1 Association of TCR clusters and cortical actin flows. (A and B) Confocal time-series images of TCR clusters (A) and EGFP-actin speckles (B) at the lamella in hybrid immunological synapse. Using particle tracking algorithms, the red track in (A) and white track in (B) represents the translocation path of TCR clusters and EGFP-actin speckles, respectively. TCR clusters behave non-diffusive motion and temporally moved along actin centripetal retrograde flow. Steady-streaming actin speckles continuously flow through and collectively direct TCR cluster translocations in a non-static manner. (C) Kymographs (time-space plot) of TCR clusters (top and red channel in the composite) and actin speckles (middle and green channel in the composite). At each time point, intensity profiles (plotted in x-axis) along the track are assembled along y-axis in time-descending order. This graphical representation reveals the spatial-tempo correlations of TCR clusters/actin speckles and confirms our previous observations. (D and E) Angle histograms of TCR cluster (red, n = 40) and actin speckle (green, n = 285) tracks, respectively. Angle of movement is derived based on the starting point along each track. The histograms suggest that TCR and actin move in nearly identical directions. Scale bar 1 µm.
(0.64 MB TIF)

Figure S2 Physical barriers on supported membrane without anti-CD3ε have no effects on changing actin dynamics. (A) Maximum projection of time-lapse images of EGFP-actin in the Jurkat T cell. (B) Supported membranes coated with labeled streptavidin, but without anti-CD3ε. (C) Kymograph along the yellow line in (A) indicates similar actin flow dynamics on both patterned and unpatterned area. The artificial effect from thin metal lines is minor and negligible.
(0.29 MB TIF)

Movie S1 TCR clusters are translocated as the actin centripetal retrograde network constantly flows from the periphery to the center of the synapse. Actin network is visualized by GFP-actin fusion protein, and TCR clusters are labeled by the anti-CD3ε on the supported lipid bilayer. Microfabricated substrate with chromium lines serves as diffusion barriers to physically restrict the lateral transport of TCR clusters. The spacing between each chromium stripe is 2 µm. The acquisition rate is 1 frame per second.
(1.78 MB MOV)

Movie S2 The transport of TCR clusters is directed by actin centripetal flow. Fluorescence speckles of GFP-actin and anti-TCR clusters are imaged by spinning-disc confocal microscopy. TCR clusters correspondingly change the direction of inward movement as actin centripetal flow remodels from the periphery to the center of the synapse. The scale bar is 1 µm, and the acquisition rate is 1 frame per second.
(2.31 MB MOV)

Movie S3 Actin centripetal retrograde flow in the spatially mutated immunological synapse. GFP-actin expressing Jurkat T cell forms the synapse over micro-patterned supported lipid bilayer (Fig. 4A inset). GFP-actin speckles are visualized by spinning-disc confocal microscopy. The scale bar is 4 µm, and the acquisition rate is 1 frame per second.
(8.11 MB MOV)
