# Supplementary figures and images for: Altered Actin Centripetal Retrograde Flow in Physically Restricted Immunological Synapses
Source: PLoS One. 2010 Jul 29;5(7):e11878. doi: 10.1371/journal.pone.0011878 (PMC2912367; doi:10.1371/journal.pone.0011878)

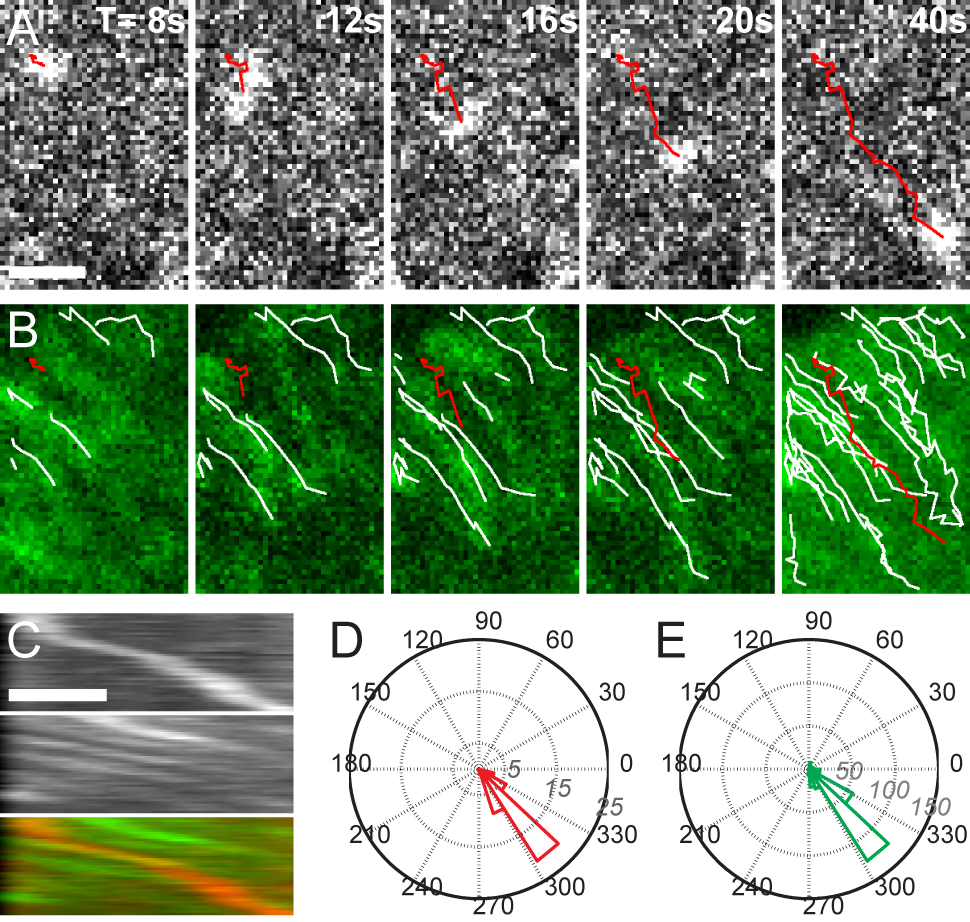

Supplement: Figure S1 — Association of TCR clusters and cortical actin flows. (A and B) Confocal time-series images of TCR clusters (A) and EGFP-actin speckles (B) at the lamella in hybrid immunological synapse. Using particle tracking algorithms, the red track in (A) and white track in (B) represents the translocation path of TCR clusters and EGFP-actin speckles, respectively. TCR clusters behave non-diffusive motion and temporally moved along actin centripetal retrograde flow. Steady-streaming actin speckles continuously flow through and collectively direct TCR cluster translocations in a non-static manner. (C) Kymographs (time-space plot) of TCR clusters (top and red channel in the composite) and actin speckles (middle and green channel in the composite). At each time point, intensity profiles (plotted in x-axis) along the track are assembled along y-axis in time-descending order. This graphical representation reveals the spatial-tempo correlations of TCR clusters/actin speckles and confirms our previous observations. (D and E) Angle histograms of TCR cluster (red, n = 40) and actin speckle (green, n = 285) tracks, respectively. Angle of movement is derived based on the starting point along each track. The histograms suggest that TCR and actin move in nearly identical directions. Scale bar 1 µm. (0.64 MB TIF) [file pone.0011878.s001.tif]

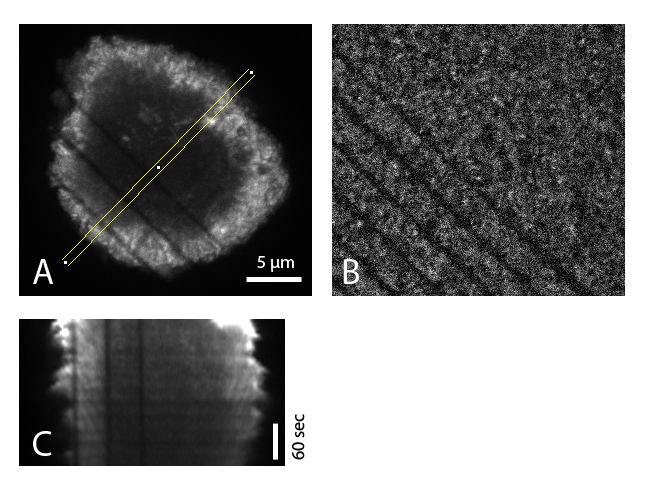

Supplement: Figure S2 — Physical barriers on supported membrane without anti-CD3ε have no effects on changing actin dynamics. (A) Maximum projection of time-lapse images of EGFP-actin in the Jurkat T cell. (B) Supported membranes coated with labeled streptavidin, but without anti-CD3ε. (C) Kymograph along the yellow line in (A) indicates similar actin flow dynamics on both patterned and unpatterned area. The artificial effect from thin metal lines is minor and negligible. (0.29 MB TIF) [file pone.0011878.s002.tif]
